# Association between dietary inflammatory index and all-cause mortality risk in adults with coronary heart disease in the United States

**DOI:** 10.1038/s41598-024-75381-6

**Published:** 2024-10-14

**Authors:** Enyang Wang, Caoyang Fang, Jing Zhang, Yuqi Wang

**Affiliations:** 1 Department of Cardiology, The Second People’s Hospital of Hefei, Hefei Hospital Affiliated to Anhui Medical University, Hefei, 230000 Anhui China; 2 Department of Emergency, First Affiliated Hospital of University of Science and Technology of China, Anhui Provincial Hospital, Hefei, 230000 Anhui China

**Keywords:** DII, Dietary inflammatory index, NHANES, All-cause mortality, CHD, Biomarkers, Cardiology, Heart failure

## Abstract

Diet and inflammation are crucial in the incidence and progression of coronary heart disease (CHD). This study aimed to investigate the correlation between the Dietary inflammatory index (DII) and all-cause mortality in CHD patients. A total of 1,303 CHD patients from the National Health and Nutrition Examination Survey (NHANES) between 2003 and 2018 were included. Multivariate Cox regression was used to explore the correlation between the DII and the risk of all-cause mortality in these patients. Restricted cubic spline (RCS) analysis was also utilized to examine the relationship between the DII and all-cause mortality risk in CHD patients. Additionally, subgroup analyses were conducted to determine how the correlation between the DII and all-cause mortality varied across different demographics. During a median follow-up period of 77 months among 1,303 CHD patients, 536 died from all causes. The DII scores were significantly higher in deceased patients compared to survivors. After adjusting for confounding factors, the multivariate Cox regression analysis indicated a strong positive correlation between the DII and all-cause mortality in CHD patients. RCS analysis suggested a non-linear relationship between the DII and all-cause mortality among CHD patients. Additionally, an increase in DII was more pronounced in its impact on female patients. The DII is strongly correlated with the risk of all-cause mortality among CHD patients, particularly among females. Thus, managing dietary inflammation is vital for the prevention and treatment of CHD, especially in female patients.

**Supplementary Information:**

The online version contains supplementary material available at 10.1038/s41598-024-75381-6.

## Introduction

Cardiovascular disease (CVD) is the principal cause of death globally^1^. Studies have demonstrated a strong correlation between the prevalence of CVD and behavioral factors, such as unhealthy diets, physical inactivity, smoking, and excessive alcohol consumption, which are preventable^2^. Diet is pivotal in preventing CVD, as evidenced by both national and international guidelines that advocate for healthy eating habits as a fundamental preventive measure^3^. A meta-analysis of randomized controlled trials has revealed that a high-quality diet is associated with a reduced risk of CVD and decreases overall mortality and cardiovascular risk^4^.

Recent research has verified that chronic low-grade inflammation significantly influences the progression of coronary artery disease and associated mortality^5^. Factors such as nutrient excess, insufficient physical activity, and aging can induce excessive secretion of inflammatory factors, leading to persistent low-grade inflammation^6^. Evidence suggests that inflammation underlies the initiation and progression of coronary artery plaques, plaque instability and rupture, and subsequent vascular remodeling and recurrent stenosis^7^. Patients exhibiting more pronounced vascular inflammatory responses have poorer prognoses. Targeted anti-inflammatory treatments have been demonstrated to diminish major adverse cardiovascular events in individuals with CHD^8,9^. Diet, a modifiable factor, is central in regulating chronic inflammation^10,11^. Previous studies on the diet-CVD nexus primarily focused on individual nutrients or specific food items, such as saturated and monounsaturated fatty acids, sodium, fruits, vegetables, meats, and plant oils. Given the complexity of dietary structures and the interaction between foods and nutrients, the concept of dietary patterns has been introduced^12^. However, most dietary patterns were not initially designed to assess dietary inflammatory properties nor fully capture an individual’s dietary inflammatory or anti-inflammatory potential. Thus, the DII was developed to evaluate the inflammatory potential of diets, first by Cavicchia et al. at the University of South Carolina in 2009 and later updated by Shivappa et al. in 2014^13,14^. They reviewed and scored 1943 studies from 1950 to 2010, creating the DII scoring algorithm based on the impact of 45 dietary components on six inflammatory markers, including interleukin-1β (IL-1β), IL-4, IL-6, IL-10, tumour necrosis factor-α (TNF-α), and C-reactive protein. A higher DII score indicates a more pro-inflammatory diet, whereas a lower score signifies a more anti-inflammatory diet, reflecting higher consumption of anti-inflammatory foods. The DII is now widely utilized in studies investigating the link between inflammation and chronic non-communicable diseases, such as CVD^15–17^, renal function^18^, and xancer^19^.

Despite extensive application, research on the correlation between the DII and all-cause mortality risk among CHD patients remains absent. This study seeks to ascertain whether a higher DII correlates with an increased risk of all-cause mortality in adults with CHD.

## Methods

### NHANES study population

**Fig. 1:**
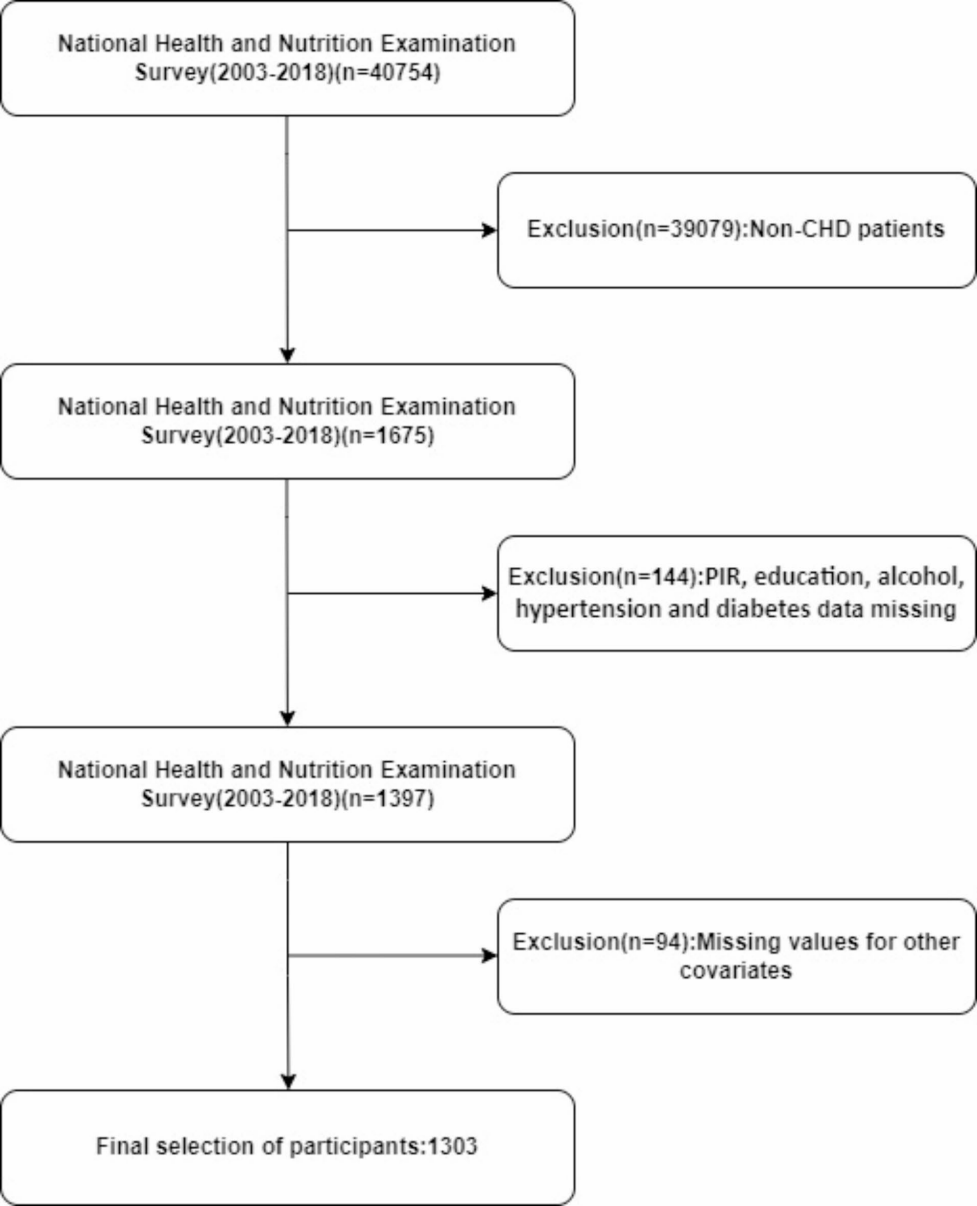
Study flow chart.

Participants were excluded based on several criteria:^1^ being under 18 years of age;^2^ missing data on DII or mortality. Additionally, those lacking information on variables such as education level, marital status, poverty-income ratio (PIR), BMI, and laboratory data were also excluded. Ultimately, 1303 eligible patients were selected. Figure 1 illustrates the specific research process. The NHANES conducts cross-sectional studies to assess the general health and nutritional status of the non-institutionalized U.S. population^20^. The NHANES protocol received approval from the Ethics Review Committee of the National Center for Health Statistics (NCHS), and all participants gave written informed consent. Given the public accessibility and the nature of NHANES data, the Ethics Committee of the Second People’s Hospital of Hefei waived the need for ethical approval for this study.

### Definition of CHD

The diagnosis of CHD relies primarily on disease history^21^. Participants confirmed their CHD status in the health questionnaire by answering whether a doctor or other health professional had diagnosed them with CHD.

### Death-related information

This study reported all-cause and CVD mortality. Mortality data from NHANES (2003–2018) were linked with death certificates from the National Death Index using a probability matching algorithm to ascertain mortality status. CVD mortality was determined by ICD-10 codes I00-I09, I11, I13, I20-I51, and I60-I69. Follow-up concluded upon the patient’s death or on December 31, 2019.

### Meal validation index

Dietary intake was recorded using the 24-hour dietary recall method, and the DII was calculated following the method developed by N. Shivappa et al.^14^. Dietary data were validated using the Nutrition Data System for Research (NDSR)^22^. In this study, 28 dietary variables were utilized to calculate the DII, including carbohydrates, protein, total fat, alcohol, fiber, cholesterol, saturated fatty acids, monounsaturated fatty acids, polyunsaturated fatty acids, n-3 fatty acids, n-6 fatty acids, niacin, vitamins A, B1, B2, B6, B12, C, D, E, and minerals such as iron, magnesium, zinc, selenium, along with folate, β-carotene, caffeine, and energy. Previous studies have demonstrated the DII’s consistent predictive ability with these 28 dietary components^23^.

### Covariates

Based on prior research and clinical experience, factors associated with CHD mortality were included: age, gender, race, educational attainment, marital status, poverty-income ratio (PIR), diabetes, hypertension, body mass index (BMI), smoking, and alcohol use. Race categories were defined as non-Hispanic black, non-Hispanic white, Mexican American, and other. PIR was categorized into three levels: < 1.0, 1.0–3.0, and > 3.0. Marital status was classified into married, divorced, unmarried, and other. Educational levels were divided into below high school, high school or equivalent, and college or higher. Smoking status was categorized into:^1^ never smokers, defined as those who smoked fewer than 100 cigarettes in their lifetime;^2^ former smokers, defined as those who smoked 100 or more cigarettes in their lifetime but no longer smoke;^3^ current smokers, who smoke on some days or daily. Alcohol intake was categorized into never, former, heavy, moderate, and mild drinkers. BMI was calculated by dividing weight (kg) by height squared (m^2^), with categories of normal (< 25 kg/m^2^), overweight (25 ≤ BMI ≤ 30 kg/m^2^), and obese (BMI > 30 kg/m^2^). Laboratory tests included Alanine aminotransferase (ALT), Aspartate aminotransferase (AST), albumin, neutrophils, lymphocytes, hemoglobin, glycated hemoglobin (HbA1c), creatinine, uric acid, and blood urea nitrogen (BUN).

### Statistical analysis

Data analysis was conducted using R software (version 4.3.2, https://www.r-project.org). The MEC sample weight (WTMEC2YR/8) was used for weighted analysis of the data. Continuous variables were presented as means (SE), and categorical variables as frequencies (%). Spearman’s rank correlation coefficient was employed to assess the correlations between continuous variables.

The relationship between the DII and all-cause mortality risk in CHD patients was explored using a multivariable Cox proportional hazards regression model. The DII was analyzed as both a continuous and categorical variable, categorized into pro-inflammatory (DII > 0) and anti-inflammatory (DII < 0) diets, to assess the robustness of the findings. Model 1 was unadjusted, Model 2 adjusted for age, race, and gender, and Model 3 included further adjustments for education level, smoking, alcohol consumption, hypertension, and diabetes, building on Model 2.

Stratified analyses were conducted based on age, gender, race, BMI, hypertension, and diabetes. To ensure the reliability of the results, we performed several sensitivity analyses. First, we excluded CHD patients who had died within the first two years of initial follow-up and reassessed the relationship between DII and all-cause mortality in CHD patients. Second, we performed non-survey-weighted multivariate logistic regression to investigate the relationship between DII and all-cause mortality in CHD patients.The relationship between the DII and all-cause mortality was also examined using RCS analysis, focusing on variations across different genders. A collinearity test was performed, considering a variance inflation factor (VIF) > 10 indicative of collinearity. All statistical methods and results were rigorously checked for accuracy and clarity.

## Results

### Baseline characteristics of participants

**Fig. 2:**
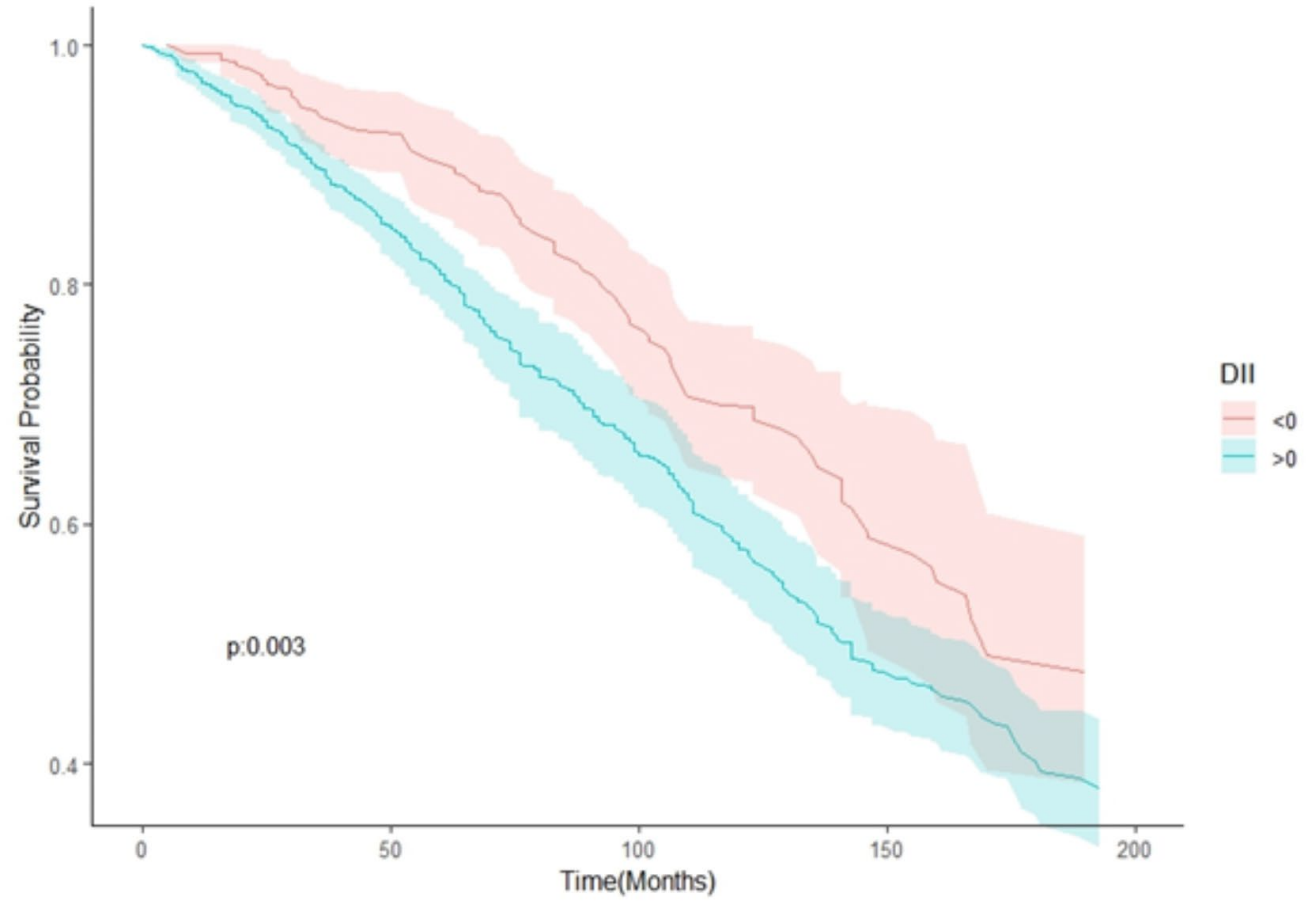
The Kaplan-Meier curve for the study population with different diets.

Table 1 presents the baseline characteristics of the CHD patients. The study included 1,303 CHD patients, with 33.95% females and an average age of 67.00 ± 0.39 years. The DII for the all-cause mortality group was significantly higher than that for the non-all-cause mortality group, with a larger proportion consuming a pro-inflammatory diet (DII > 0). Kaplan-Meier survival curves, as depicted in Fig. 2 and stratified by DII > 0 and DII < 0, demonstrated that CHD patients consuming a pro-inflammatory diet exhibited a poorer prognosis (log-rank test, *p* < 0.05). Figure S1 displays the Spearman rank correlation coefficient for continuous variables, revealing a high or moderate correlation among some variables.

**Table 1:** Baseline characteristics of patients.

Variables	Total	Survival group	Death group	*P*-value
Age, years, mean (SE)	67.00 (0.39)	64.14 (0.44)	72.50 (0.46)	< 0.0001
HbA1c, (%)	6.17 (0.04)	6.08 (0.05)	6.34 (0.08)	0.01
ALT, IU/L, mean (SE)	25.83 (1.09)	25.92 (0.96)	25.65 (2.66)	0.93
AST, IU/L, mean (SE)	26.66 (0.58)	26.07 (0.60)	27.81 (1.22)	0.21
Albumin, g/dL, mean (SE)	4.17 (0.01)	4.19 (0.01)	4.12 (0.02)	< 0.001
Creatinine, umol/L, mean (SE)	96.13 (1.40)	90.87 (1.61)	106.23 (2.49)	< 0.0001
Uric acid, umol/L, mean (SE)	360.00 (3.40)	350.87 (4.50)	377.57 (5.51)	< 0.001
BUN, mmol/L, mean (SE)	6.30 (0.11)	5.76 (0.11)	7.35 (0.19)	< 0.0001
Neutrophils, ×10^9^/L, mean (SE)	4.59 (0.06)	4.49 (0.07)	4.79 (0.11)	0.03
Lymphocytes, × 10^9^/L, mean (SE)	2.12 (0.14)	2.23 (0.20)	1.92 (0.08)	0.16
Hemoglobin, g/dL, mean (SE)	14.17 (0.06)	14.32 (0.07)	13.89 (0.09)	< 0.0001
BMI, n(%)				
< 25	19.93 (0.02)	18.02 (1.92)	23.58 (1.89)	< 0.0001
25–30	33.71 (0.02)	32.99 (1.99)	35.08 (2.57)	
> 30	46.37 (0.03)	48.98 (2.55)	41.34 (2.86)	
Sex, n(%)				
Male	66.05 (0.04)	65.89 (2.48)	66.35 (2.18)	0.9
Female	33.95 (0.03)	34.11 (2.48)	33.65 (2.18)	
Race, n(%)				
Mexican American	3.25 (0.03)	3.85 (2.48)	2.08 (0.59)	0.06
Non-Hispanic Black	5.5 (0.01)	6.24 (0.7)	4.07 (0.73)	
Non-Hispanic White	83.21 (0.05)	81.05 (1.71)	87.38 (1.77)	
Other	8.04 (0.01)	8.86 (1.34)	6.47 (1.53)	
Marital, n(%)				
Married	63.79 (0.04)	66.68 (2.43)	58.22 (2.71)	< 0.001
Never married	4.97 (0.01)	5.95 (1.27)	3.09 (0.93)	
Divorced	10.51 (0.01)	10.84 (1.42)	9.86 (1.29)	
Unmarried but have/had partner	20.73 (0.02)	16.53 (1.9)	28.83 (2.22)	
Education, n(%)				
Less than high school	21.7 (0.02)	18.67 (1.69)	27.54 (2.65)	< 0.001
High school or equivalent	26.98 (0.03)	25.31 (2.18)	30.19 (2.41)	
College or above	51.32 (0.03)	56.02 (2.41)	42.27 (2.67)	
Smoke, n(%)				
Never	34.96 (0.02)	37.15 (2.25)	30.75 (1.98)	0.003
Former	47.18 (0.03)	43.17 (2.43)	54.88 (2.32)	
Now	17.86 (0.02)	19.68 (2.1)	14.37 (2.03)	
Alcohol, n(%)				
Never	10.5 (0.01)	9.06 (1.3)	13.27 (1.74)	< 0.0001
Former	28.33 (0.02)	22.19 (1.98)	40.16 (2.99)	
Mild	42.66 (0.03)	45.62 (2.62)	39.96 (2.64)	
Moderate	9.19 (0.01)	11.71 (1.49)	4.34 (1.12)	
Heavy	9.32 (0.01)	11.43 (1.59)	5.26 (0.96)	
Diabetes, n(%)				
Yes	41.69 (0.03)	39.26 (2.16)	46.39 (2.42)	0.01
No	46.63 (0.03)	50.13 (2.51)	39.91 (2.42)	
Borderline	11.67 (0.01)	10.61 (1.36)	13.71 (1.54)	
Hypertension, n(%)				
Yes	76.49 (0.04)	74.18 (2.11)	80.93 (1.97)	0.03
No	23.51 (0.02)	25.82 (2.11)	19.07 (1.97)	
PIR, n(%)				
< 1	19.93 (0.02)	18.02 (1.92)	23.58 (1.89)	0.07
1–3	33.71 (0.02)	32.99 (1.99)	35.08 (2.57)	
> 3	46.37 (0.03)	48.98 (2.55)	41.34 (2.86)	
DII	1.51 (0.06)	1.43 (0.09)	1.69 (0.08)	0.03
DII (> 0, < 0)				
< 0	22.53 (0.02)	25.84 (2.26)	16.14 (1.99)	0.001
> 0	77.47 (0.04)	74.16 (2.26)	83.86 (1.99)	

Date are presented as mean (SE) or n (%).

### Association between DII and all-cause mortality in CHD patients

**Fig. 3:**
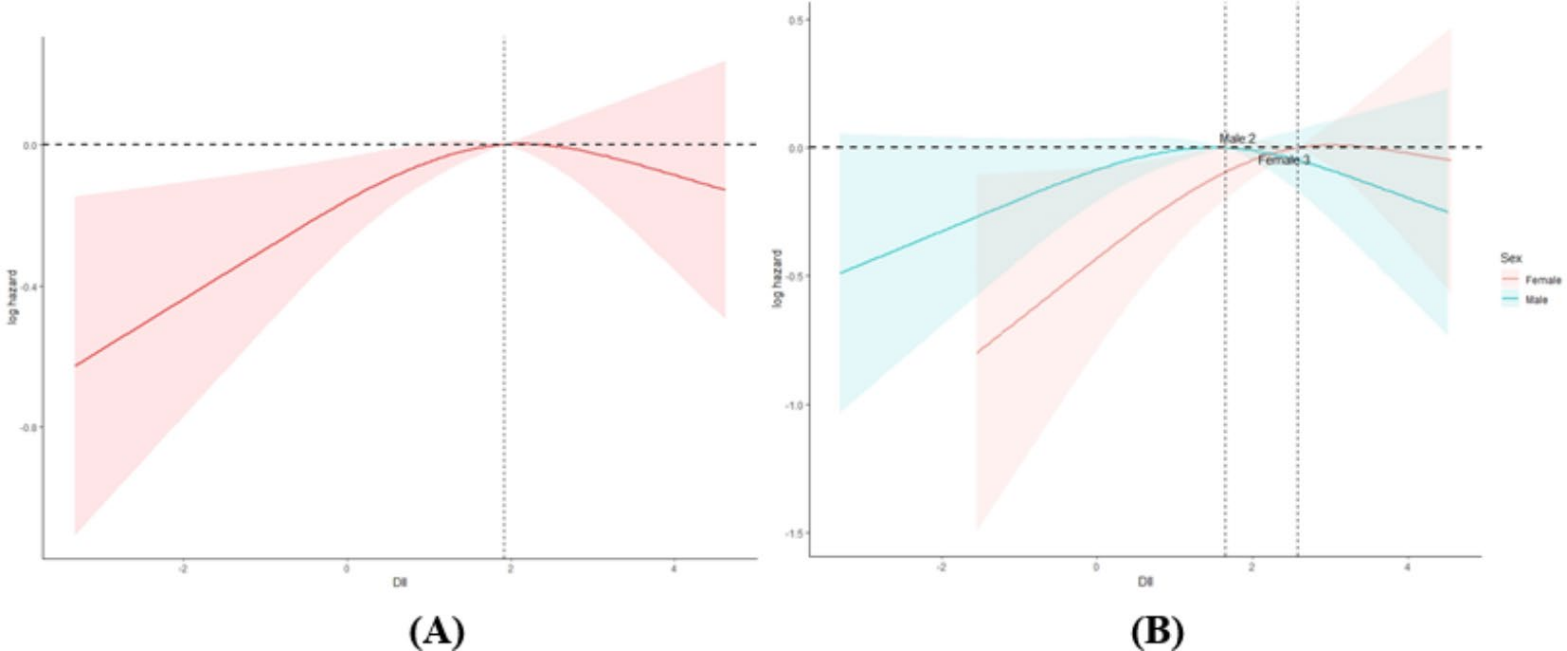
RCS analysis of the association between DII and all-cause mortality in coronary heart disease. (**A**) RCS curves of DII and risk of all-cause mortality from coronary heart disease in all subjects, (**B**) RCS curves of DII and risk of all-cause mortality from coronary heart disease in male and female subjects.

Of the 1,303 CHD patients monitored, 536 succumbed to all causes. RCS analysis indicated a non-linear relationship between DII and all-cause mortality among CHD patients (Fig. 3A). As shown in Table 2, after adjusting for multiple variables, a higher DII was associated with an increased risk of all-cause mortality in CHD patients.

**Table 2:** Association between DII and all-cause mortality.

Variables	Model 1		Model 2		Model 3	
	HR (95% CI)	*P*	HR (95% CI)	*P*	HR (95% CI)	*P*
DII	1.06 (1.01, 1.12)	0.02	1.09 (1.07, 1.1)	< 0.0001	1.06 (1, 1.12)	0.05
DII(> 0, < 0)						
< 0	Ref	Ref	Ref	Ref	Ref	Ref
> 0	1.51 (1.15, 1.99)	0.003	1.53 (1.16, 2.02)	0.002	1.41 (1.05, 1.9)	0.02

*HR* hazard ratio,* CI* confidence interval,* Ref* reference.

### Subgroup analysis

**Fig. 4:**
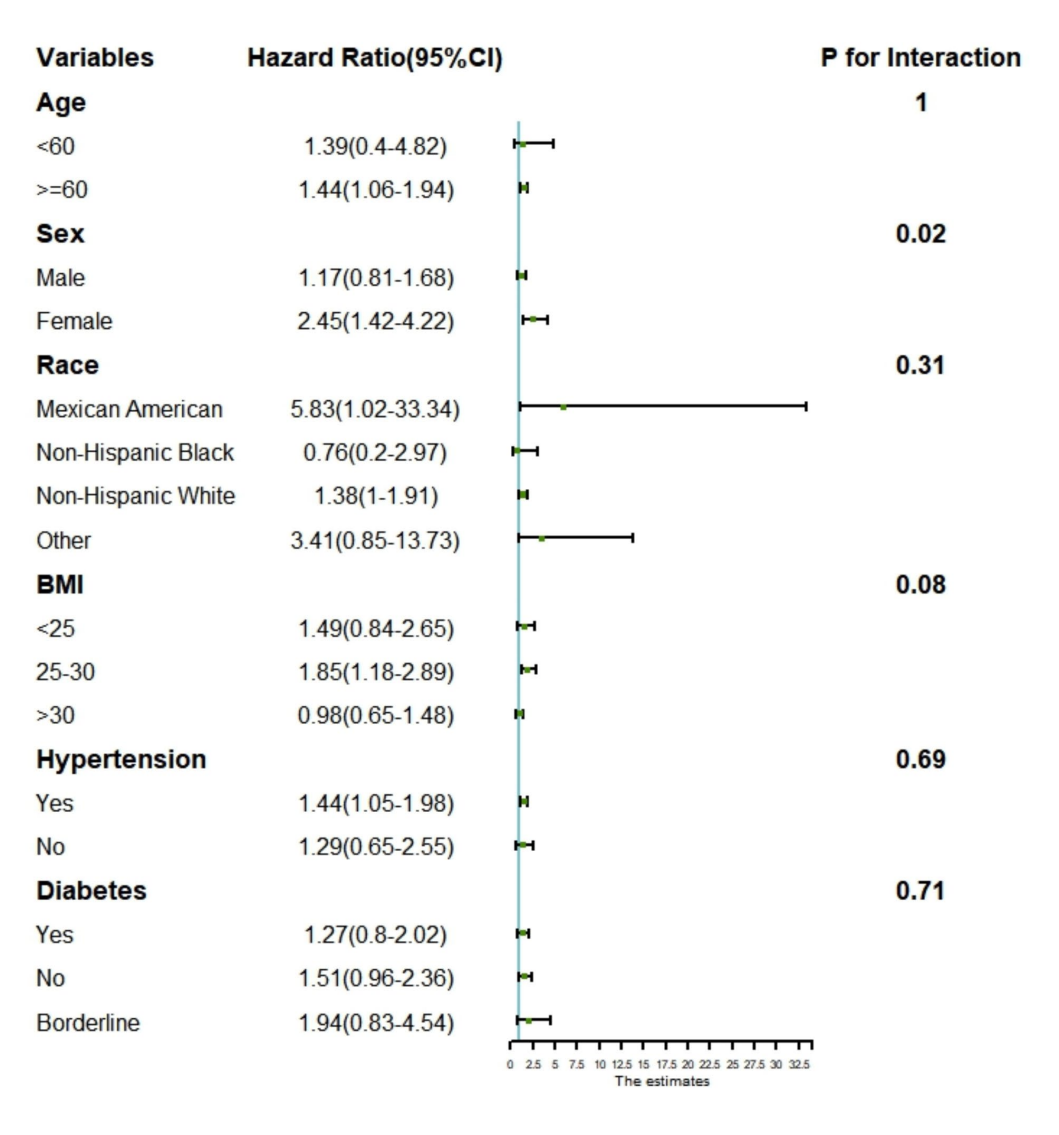
Stratified analysis of relationship between DII and risk of all-cause mortality in patients with coronary heart disease.

Subgroup analysis depicted in Fig. 4 revealed variations in the association between DII and all-cause mortality across different strata such as age, gender, race, BMI, smoking status, hypertension, and diabetes, with significant differences noted by gender (*P* < 0.05). Notably, for women, each unit increase in DII significantly heightened the risk of all-cause mortality. Figure 3B further highlighted that the impact of an increasing DII was more pronounced among females.

### Sensitivity analysis

First, the single and combined impact of all-cause mortality was essentially the same in DII versus CHD patients after excluding death events in the first two years (see Table S1 for details). Second, the weighted results showed slightly smaller OR compared to the unweighted results, but overall maintained consistency associated with statistical significance (see Table S2 for details). In addition, due to the particularity of NHANES data samples, the weighted adjusted results are more accurate.

## Discussion

Our analysis of the association between DII and the likelihood of all-cause mortality in CHD patients yielded several key findings:^1^ CHD patients who died from all causes had a significantly higher DII compared to those who survived;^2^ the relationship between DII and all-cause mortality risk in CHD patients was non-linear and showed a positive correlation;^3^ compared to pro-inflammatory diets, anti-inflammatory diets were associated with a reduced risk of mortality from all causes in CHD patients; and^4^ female CHD patients demonstrated a greater sensitivity to DII compared to male CHD patients. Given the cross-sectional nature of this study, these results are correlational. Further prospective studies are necessary to elucidate the relationship between DII and all-cause mortality risk in CHD patients.

Chronic inflammation is a primary trigger for chronic non-communicable diseases, including CVDs. Over-nutrition and lack of exercise may lead to an overproduction of cellular inflammatory factors^24,25^. CHD, characterized as a chronic inflammatory condition, involves multiple pathogenic factors that induce atherosclerosis in arteries throughout the body. This progressive and cumulative process serves as the main pathological foundation for CHD development, gradually resulting in narrowing or complete occlusion of the vascular lumen. Such changes lead to inadequate oxygen and blood supply to the vessel, potentially causing ischemia, edema, and necrosis of myocardial cells. Inflammatory variables like high-sensitivity C-reactive protein, IL-6, IL-8, and IL-1β are potent mediators in CHD progression and are predictive of disease severity^26^. Various theories explain CHD mechanisms from different perspectives. Substantial evidence indicates that inflammation is integral to every stage of coronary artery atherosclerosis development^27^. In prospective cohort studies, elevated levels of inflammatory markers—including IL-6, C-reactive protein, tumor necrosis factor-α, and soluble cell adhesion molecule 1—are positively correlated with the occurrence of adverse cardiovascular events. Both randomized trials and Mendelian randomization studies support the causal role of inflammatory cytokines such as IL-1β and IL-6 in CVD onset^28,29^. Diet, as a significant modifiable risk factor, is linked to CVD incidence and influences inflammation^30^. Additionally, numerous studies have demonstrated a correlation between diets rich in dietary fiber, long-chain n-3 polyunsaturated fatty acids, and antioxidants and lower levels of inflammatory markers^31,32^. Therefore, diet significantly influences CVD development, primarily through its regulatory effect on inflammation.

However, to date, no study has investigated the relationship between all-cause mortality risk and DII in individuals with CHD. The current research revealed that CHD patients who died from all causes had significantly higher DII levels. Dietary factors are crucial in the prevention and management of CHD. Previous research has indicated that increased consumption of vegetables, fruits, nuts, fish, and vitamin A, C, E, as well as folate and dietary fiber, may reduce the risk and progression of CHD^33,34^. Conversely, higher intake of cholesterol, eggs, red meat, processed meat, and sugary drinks may increase both the risk and progression of CHD. A cross-sectional study of 204,802 Chinese adults showed that higher sodium intake and lower fruit consumption were associated with an increased number of cardiovascular metabolic disease-related deaths^35^. The University of South Carolina developed the DII to assess the inflammatory potential of an individual’s diet. Numerous studies have examined the relationship between DII and the etiology of chronic diseases, particularly CVDs. A study by Lana M. Agraib et al. in the Jordanian population found that higher DII scores were positively correlated with an increased risk of CHD, with each unit increase in DII scores associated with a 1.13-fold increase in CHD risk. Similar findings have been reported in populations in the United States and Australia, showing positive correlations between higher DII scores and CHD risk. Although many studies have established a positive correlation between inflammation and CHD risk, none have explored the relationship between dietary inflammatory potential and all-cause mortality risk in CHD patients. According to our results, in fully adjusted models, there was a positive correlation between DII and the risk of all-cause mortality (OR = 1.06, 95% CI: 1-1.12, *P* > 0.05), with CHD patients on pro-inflammatory diets (DII > 0) experiencing a 41% higher risk of all-cause mortality compared to those on anti-inflammatory diets (DII < 0). This suggests that dietary inflammation is positively associated with all-cause mortality risk in CHD patients, with higher dietary inflammation potentially increasing the risk and more anti-inflammatory diets potentially reducing it.

The RCS analysis indicated a non-linear relationship between DII and all-cause mortality risk in CHD patients. Kaplan-Meier survival curves also demonstrated a higher risk of all-cause mortality among CHD patients consuming pro-inflammatory diets (DII > 0). A recent large-scale prospective study, which followed 155,724 individuals from 21 countries over approximately ten years, investigated the relationship between dietary quality and CVD. The primary outcome was a composite of major cardiovascular events, including heart failure, myocardial infarction, stroke, and cardiovascular mortality. The study found that the correlation between poor dietary quality and CVDs was stronger in women than in men, with likelihood ratios of 1.17 (95% CI: 1.08–1.26) and 1.07 (95% CI: 0.99–1.15), respectively^36^. Similar to these findings, our study also revealed a greater risk of all-cause mortality among female CHD patients compared to male patients, with risk ratios of 2.45 (95% CI: 1.42–4.22) and 1.17 (95% CI: 0.81–1.68), respectively. Consequently, our research suggests that utilizing DII to assess nutritional quality may reduce the risk of all-cause mortality among female CHD patients.

This study has several limitations. Firstly, as a cross-sectional study, it cannot establish causal relationships. Despite efforts to control for various potential confounders, residual confounding effects on the DII cannot be completely eliminated. Future prospective randomized controlled studies are required to determine the causal relationship between DII and all-cause mortality risk in patients with CHD. Secondly, the DII, being a composite index, may not encompass all aspects of diet-related inflammation, particularly when relying on self-reported data, which may introduce recall bias and misclassification. Additional prospective randomized controlled studies are necessary to validate these results. Finally, the study’s findings, based exclusively on American participants, do not readily generalize to other populations. Further multicenter randomized controlled trials across various countries and regions are essential to determine if these conclusions are applicable to different demographic groups.

## Conclusions

In conclusion, this study underscores the importance of dietary management in CHD patients. Evaluating the DII can assist in adjusting dietary structure, balance nutrition, and enhancing quality of life. Moreover, it offers crucial insights for the early prevention of CHD and strategies to mitigate all-cause mortality risk through dietary control, particularly in female CHD patients. The study also provides valuable data for future international research on this topic.

## Electronic supplementary material

Below is the link to the electronic supplementary material.

Supplementary Material 1

## Data Availability

The datasets used and/or analysed during the current study available from the corresponding author on reasonable request.
