# Supplementary material for: Association between dietary inflammatory index and all-cause mortality risk in adults with coronary heart disease in the United States
Source: Sci Rep. 2024 Oct 14;14:23998. doi: 10.1038/s41598-024-75381-6 (PMC11473697; doi:10.1038/s41598-024-75381-6)
Supplement: Supplementary file 1 — Supplementary Material 1 [file 41598_2024_75381_MOESM1_ESM.doc]

**Table S1.Association between DII and all-cause mortality in patients with CHD: Patients with CHD who developed death within the first two years of initial follow-up were excluded**

| **Variables** | **Model 1** | | **Model 2** | | **Model 3** | |
| --- | --- | --- | --- | --- | --- | --- |
|  | **HR(95%CI)** | **P** | **HR(95%CI)** | **P** | **HR(95%CI)** | **P** |
| **DII** | 1.05(1,1.11) | 0.07 | 1.09(1.03,1.16) | 0.003 | 1.06(1,1.13) | 0.06 |
| **DII(>0,<0)** | | | | | | |
| <0 | Ref | Ref | Ref | Ref | Ref | Ref |
| >0 | 1.41(1.05,1.9) | 0.02 | 1.6(1.22,2.1) | <0.001 | 1.47(1.1,1.97) | 0.01 |

**Model adjusted for sex, age, race, education, smoking, alcohol consumption, BMI, hypertension, diabetes**

**Table S2.Association between DII and all-cause mortality in patients with CHD: non-survey weighted multivariate Cox regression**

| **Variables** | **Model 1** | | **Model 2** | | **Model 3** | |
| --- | --- | --- | --- | --- | --- | --- |
|  | **HR(95%CI)** | **P** | **HR(95%CI)** | **P** | **HR(95%CI)** | **P** |
| **DII** | 1.02(0.97,1.07) | 0.51 | 1.06(1.01,1.12) | 0.02 | 1.04(0.98,1.1) | 0.2 |
| **DII(>0,<0)** | | | | | | |
| <0 | Ref | Ref | Ref | Ref | Ref | Ref |
| >0 | 1.2(0.94,1.53) | 0.15 | 1.39(1.08,1.78) | 0.01 | 1.3(1.01,1.67) | 0.04 |

**Model adjusted for sex, age, race, education, smoking, alcohol consumption, BMI, hypertension, diabetes**

**
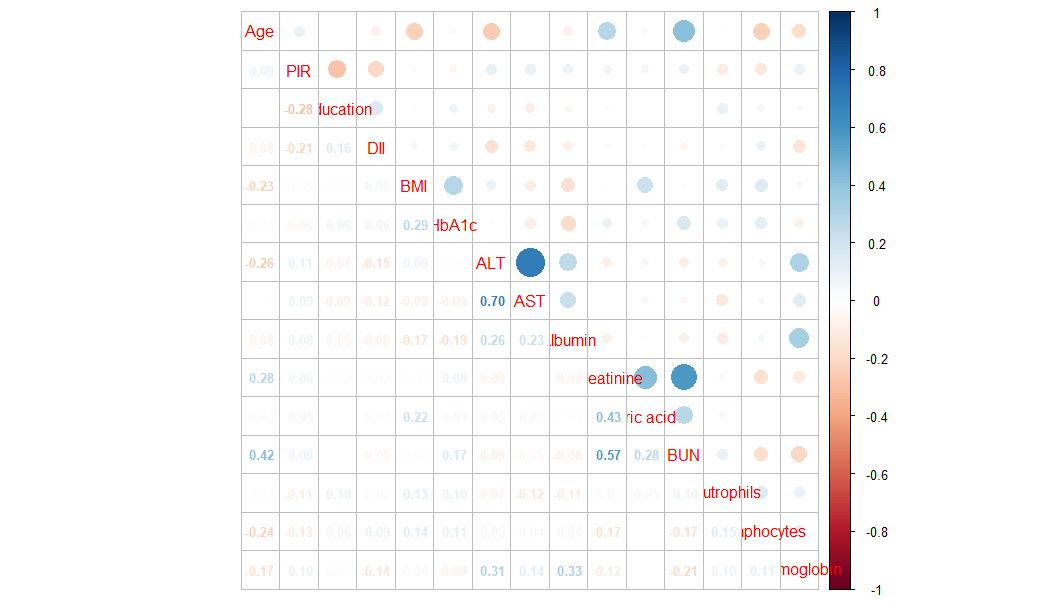
**

**Figure S1.Correlation of continuous variables**
